# 

**Published:** 1864-03

**Authors:** 


### CHICAGO MEDICAL JOURNAL.

☞ Business letters to the Journal should be addressed to Drs. Miller & Ingals, Chicago, Ills., P. O. Drawer 5787. When money is received a receipt will be returned with the next number of the Journal, and subscribers failing to get such receipt will confer a favor by giving us notice of any omission.

Vaccine Matter.—Carefully selected Vaccine Matter may be obtained by enclosing One Dollar and addressing *Chicago Medical Journal*, P. O. Drawer 5787, Chicago, Ills.

